# A Simple 2D Non-Parametric Resampling Statistical Approach to Assess Confidence in Species Identification in DNA Barcoding—An Alternative to Likelihood and Bayesian Approaches

**DOI:** 10.1371/journal.pone.0050831

**Published:** 2012-12-11

**Authors:** Qian Jin, Li-Jun He, Ai-Bing Zhang

**Affiliations:** 1 College of Life Sciences, Capital Normal University, Beijing, P. R. China; 2 State Key Laboratory of Estuarine and Coastal Research, East China Normal University, Shanghai, P. R. China; Biodiversity Insitute of Ontario - University of Guelph, Canada

## Abstract

In the recent worldwide campaign for the global biodiversity inventory via DNA barcoding, a simple and easily used measure of confidence for assigning sequences to species in DNA barcoding has not been established so far, although the likelihood ratio test and the Bayesian approach had been proposed to address this issue from a statistical point of view. The TDR (Two Dimensional non-parametric Resampling) measure newly proposed in this study offers users a simple and easy approach to evaluate the confidence of species membership in DNA barcoding projects. We assessed the validity and robustness of the TDR approach using datasets simulated under coalescent models, and an empirical dataset, and found that TDR measure is very robust in assessing species membership of DNA barcoding. In contrast to the likelihood ratio test and Bayesian approach, the TDR method stands out due to simplicity in both concepts and calculations, with little in the way of restrictive population genetic assumptions. To implement this approach we have developed a computer program package (TDR1.0beta) freely available from ftp://202.204.209.200/education/video/TDR1.0beta.rar.

## Introduction

DNA barcoding (http://www.barcodinglife.org) gained widespread prominence during the past nine years as part of the worldwide campaign for global biodiversity inventory [1]–[15] although reservations remain [16]–[24]. Most of empirical DNA barcoding studies have applied tree-based methods (NJ, MP, Bayesian) or BP-based method for the assignment of sequences to known species [12]. These methods performed reasonably well in species identification over a wide range of evolutionary scenarios [12], [25].

With the increasing of empirical studies of DNA barcoding, theoretical biologists have provided several methods with statistical point of view, e.g., a likelihood ratio test and a Bayesian approach for estimating the rate of correct specimen assignment under DNA barcoding [26], [27]. Compared to the Bayesian approach, the likelihood ratio test was shown to have less power in that it requires more assumptions [27]. The Bayesian approach [27] relies on rather restrictive population genetic assumptions, such as constant population size or no migration between two populations (to model species divergence). Furthermore, the existing measures of statistical confidence in specimen identification using DNA barcoding require strong assumptions regarding the population genetics of the species involved [27]. Clearly, such restrictive assumptions render problematic the use of DNA barcoding for specimen identification in biodiversity assessment. Measures which do not require strong assumptions about the population genetics of target species are highly desirable.

We here propose a new and simple measure of confidence of species membership - the TDR value (Two Dimensional non-parametric Resampling) which has the advantages of conceptual simplicity, computational feasibility, and minimally restrictive population genetic assumptions, as an alternative to the likelihood and Bayesian Approaches. Non-parametric resampling approaches are often simpler and more accurate, require fewer assumptions, and have greater generalizability, when compared to standard methods of statistical inferences [28]. The TDR approach suggested in this study is superior to currently existing approaches in that: (1) it does not require an estimation of phylogenetic relationships and (2) it provides a measure of confidence in barcoding even in the absence of the species in the database. Below we develop this new measure of confidence in specimen identification and assess its general validity and robustness using both data simulated under coalescent models, and an empirical data [2] from the skipper butterflies of the *Astraptes fulgerator* complex. We also use the butterfly data to compare the new TDR method with the Bayesian method [27] in terms of power to make sequence identification.

## Results

### Evaluating The New Measure Of Confidence (TDR) With Coalescent Simulation Datasets And Empirical Datasets

To evaluate the new measure of confidence (TDR) defined here, we used both computer simulated and empirical datasets. Theoretically, TDR values can range from 0 to 100%, with higher TDR values indicating higher confidence. Query sequences which are actually sampled from the same species (intraspecific query) are expected to generate higher TDR values than query sequences which are sampled from different species (interspecific query). Also randomly generated DNA query sequences (random query) should produce lower TDR values than query sequences from the same or closely related species.

#### Simulated data sets

Simulations were performed using Mesquite version 1.12 [29]. First, 10 species trees, each of eight species, were randomly generated by pure birth processes using Mesquite's Uniform Speciation (Yule) module (Appendix S1). Within each species tree, coalescent simulations were performed to generate gene trees. We considered 4 tree depths from shallower species trees to deeper ones 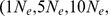 and 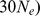 with two different effective population sizes of 100,000 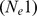 and 1,000,000 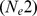. Using this strategy we attempt to address variation in gene coalescence depths within species relative to the level of genetic divergence between species. Examples of gene trees contained in species trees are shown in Appendix S1b. We then simulated sequence evolution along those gene trees to generate a set of 36 sequences per species for each replicate. The length of the sequence was fixed to 648 base pairs, which is usual for DNA barcoding using the cox1 mitochondrial gene [1], [2],Hebertetal2003b. For simplicity, coalescent simulations were performed only with the 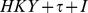 model [30]. We set the transition/transversion ratio 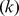 equal to 3, the discrete gamma parameter 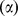 to 0.5 with 4 categories, proportion of invariable sites to 0.26, and the frequencies of nucleotides A, C, G, and T, to 0.28, 0.15, 0.25 and 0.32, respectively, derived from those studies [12], [31]. To achieve an appropriate level of overall sequence divergence, we applied an internal Mesquite scaling factor of 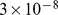. Based on settings above, 80 DNA sequence matrices were generated, each containing 288 sequences. In addition, we further extended the transition/transversion ratio 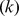 to 0.5 and 13, the discrete gamma parameter 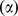 to 0.2 and 20, to examine the effect of different settings of parameters on the calculations of interspecific and intraspecific TDR values. We then randomly selected 11 sequences (about one third) from each of eight species as query data. The remaining 25 sequences (about two thirds) per species were used as reference sequences. Intraspecific and interspecific TDR values were calculated according to formula (1) for each query sequence. Mean TDR values over all query sequences of each species on each species tree were also calculated (Appendix S2). Table 1 shows the TDR values for inter- and intraspecific comparisons over all simulations (10 species trees, 4 tree depths and two effective population sizes). In the case of shallower species (depth = 1 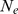, Table 1), the mean TDR values of intraspecific queries reached 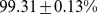
(SE) for 1
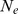
1 and 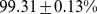 (SE) 1 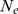
2, which are very close to the theoretical upper limits (1.00) or 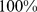 while the average TDR values of interspecific queries were 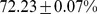 for 1 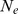
1 and 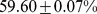 for 1 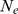
2 although a few interspecific queries yielded high TDR values. This indicates the risk of false positves in species identification via DNA barcoding when closely related species with incomplete lineage sorting are included (Appendix S1b). In such a case, we caution that the standard short barcode sequence is not sufficient for species identification. We propose the use of longer fragments or several loci as suggested earlier [12], [32]. In the situation of the 5 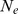 tree depth, the mean TDR values of intraspecific and interspecific queries presented similar pattern as in 1 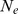 tree depth, but with relatively low risk of false identification (Table 1, see also Appendix S1b). With even deeper tree depths and longer evolutionary histories (10 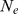 and 30 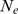, Table 1, see also Appendix S1b), the mean TDR values of interspecific queries decreased further (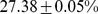 for 10 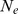
1; 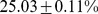 for 10 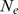
2; 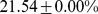 for 30
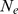
1; 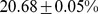 for 30 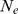
2), indicating an even lower risk of false positives in species identification than in the situations of 1 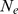 and 5 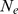. The average TDR values of intraspecific queries were as high as theoretically expected (for example, 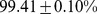
(1 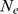
1), 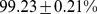 (10 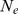
2), 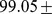
0.17% (30 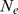
1), 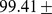
0.10%(30 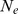
2);Table 1; see also Appendix S2 for the results of the simulations on each species tree). Varing the settings of parameters, such as, the transersion/transversion ratio (taking two more values of 0.5 and 13), and gamma parameter (two more values of 0.2 and 20), did not change the pattern of intraspecific and intespecific TDR values, reflecting the robustness of the TDR method (see Appendix S3).

**Table 1:** Average intraspecific and interspecific TDR values (%) among eight coalescent simulated species evolved on species trees with different tree depths (1 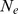, 5 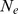, 10 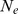, and 30 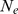,) and effective population sizes of 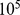 (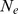
1) and 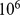 (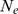
2) over ten species trees.

Tree	Ne	Intraspecific		Interspecific	
depth		TDR 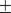 SE (K2P)	TDR 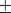 SE (HKY)	TDR 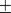 SE (K2P)	TDR 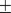 SE (HKY)
1Ne	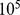	99.30 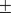 0.12	99.43 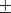 0.09	72.23 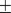 0.06	72.29 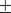 0.05
	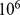	99.30 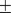 0.12	99.14 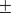 0.16	59.59 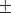 0.06	59.20 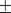 0.08
5Ne	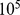	99.16 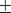 0.14	99.22 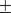 0.14	31.93 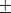 0.07	31.88 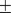 0.07
	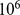	99.52 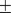 0.14	99.41 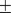 0.12	27.33 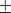 0.07	27.63 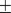 0.06
10Ne	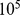	99.41 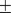 0.09	99.30 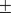 0.12	27.37 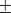 0.05	27.45 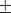 0.06
	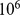	99.23 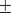 0.21	99.37 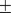 0.14	25.02 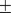 0.11	25.31 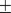 0.07
30Ne	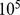	99.04 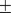 0.17	99.30 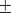 0.12	21.53 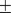 0.09	21.35 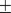 0.06
	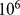	99.40 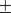 0.09	99.29 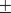 0.14	20.68 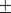 0.05	20.47 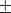 0.07

**Figure 1:**
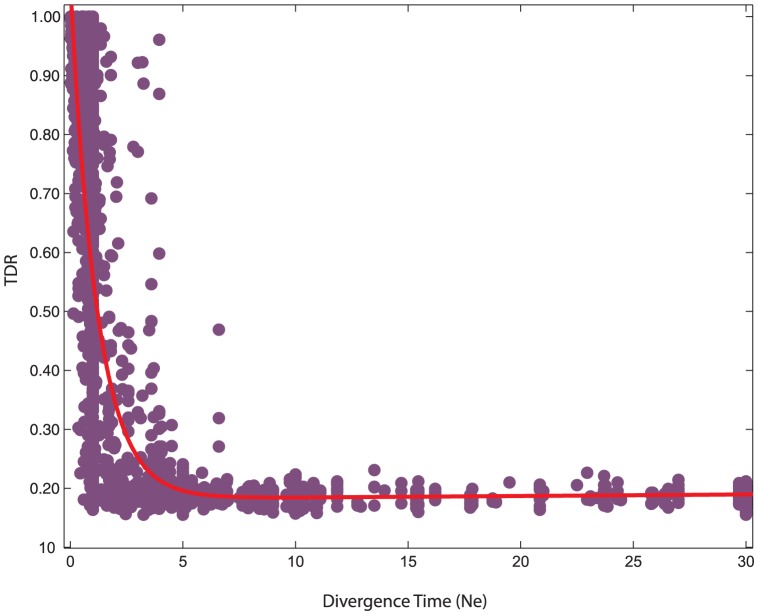
Outline of the two-dimensional non-parameter resampling approach used in this study. Both reference sequences (Ref) and query sequence (Que) are pooled together. First, a horizontally resampling over sites was performed to generate sequence matrices (R1Rn), such as R1. Second, a vertical random permutation over the matrices R1Rn was performed by randomly designating a sequence as the query sequence, the remaining sequences as references, with such a vertical permutation being repeated 100 times per R matrix. In each random permutation, the mean genetic distance between the randomly selected query sequence and the remaining reference sequences was calculated. Third, the null hypothesis that the reference sequences and query sequence resampled belong to the same species was tested (the null hypothesis is accepted if the observed genetic distance (GD) falls into the acceptance area of 95% given the simulated datasets, and is otherwise rejected). Fourth, the number of cases where the null hypothesis was accepted over all 100 replications of horizontally resampling is counted, and is the TDR measure defined in this study (see text for details).

To explore the relationship between TDR values and species divergence time, we plotted TDR values from all evolutionary scenarios versus species divergence time (Fig. 1). The divergence time was scaled by effective population size and intraspecific comparisons were not considered. TDR values dramatically decreased with increasing divergence time, especially when species divergence time is large than 5 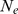 (Fig. 1).

#### Comparison of the TDR approach with a Bayesian one using the butterfly data

**Figure 2:**
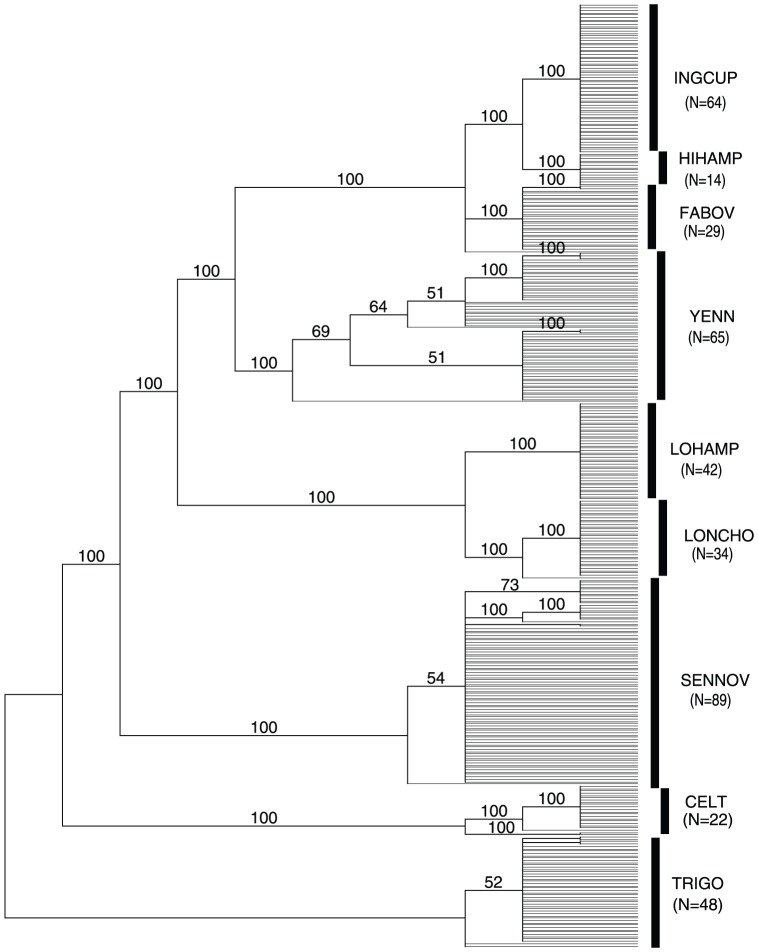
Relationship between TDR values and divergence time for interspecific comparisons. The results presented in this figures are summarized from 10 species trees with two different population sizes of 100,000 (1) and 1,000,000 (2). The red line is a fitted curve of tendency.

**Figure 3:**
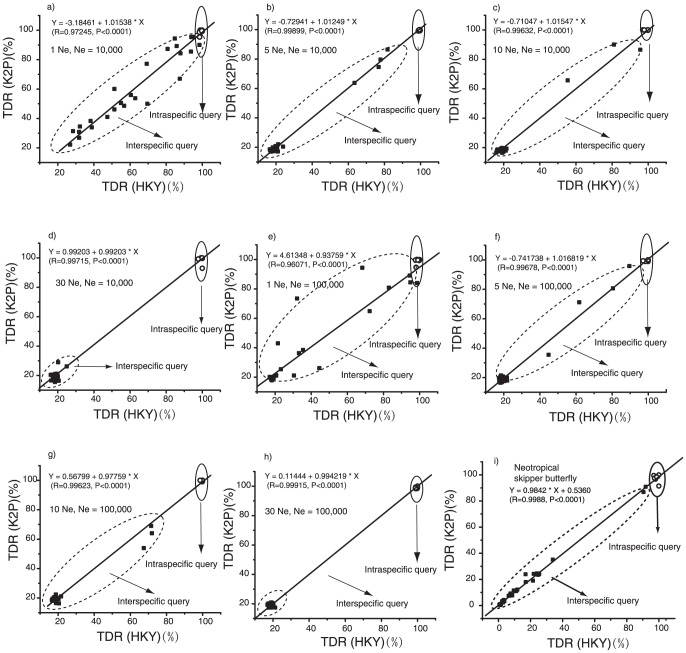
A majority consensus of 1979 MP trees of Neotropical skipper butterfly. Species encodings were taken from Hebert et al. (2004). The values above each branch are bootstrap values (not shown for less than 50).

We also studied an empirical data set from the Neotropical skipper butterfly “*Astraptes fulgerator*” (Lepidoptera: Hesperiidae), which recently has been proposed to form a complex of at least 10 separate species on the basis of DNA barcoding and other attributes [33], but see [19] for an opposing view. The 407 mitochondrial COI sequences of Astraptes fulgerator were obtained from the published DNA barcoding project (Code-EPAF: http://barcodinglife.org/views/projectlist.php?&). We removed sequences which were too short or contained ambiguous characters. The remaining sequences were aligned using ClustalX version 1.83 [34], resulting in an alignment of 630 bp [12]. The phylogenetic relationships of sequences and taxa are shown in Fig. 2. As we did with the simulated datasets, we randomly selected two thirds of the sequences from each of nine species as reference sequences. The remaining one third of the sequences of each species were used as query sequences. We calculated TDR values according to formula (1) for each query sequence within species and between species (Table 2, see also Fig. 3i). The results show that the intraspecific queries achieved very high mean TDR values (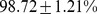) over all species studied while interspecific queries obtained relatively low mean TDR values (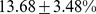). The result indicates that we can have very high confidence in assigning these query sequences to their correct species. However, caution should still be taken for the cases of low intraspecific sampling where TDR values could be overestimated and the risk of false positives may increase.

**Table 2:** Pair-wise TDR values (%) among nine Neotropical skipper butterfly species “*Astraptes fulgerator*” (Lepidoptera: Hesperiidae).

	CE	FAB	HIH	ING	LOH	LON	SEN	TRI	YE
	LT	OV	AMP	CUP	AMP	CHO	NOV	GO	NN
	**100.0**	33.86	90.43	2.29	11.00	25.43	1.00	6.86	2.29
CELT	0.00	**99.33**	91.78	16.89	11.33	22.00	1.22	8.33	3.67
FABOV	1.39	0.47	**100.0**	17.00	9.50	21.50	0.50	7.50	3.25
HIHAMP	0.81	1.04	0.00	**97.90**	11.76	24.71	1.57	7.19	2.43
INGCUP	0.47	2.57	0.82	2.09	**100.0**	24.08	0.77	6.38	2.38
LOHAMP	1.54	0.90	1.50	0.81	0.00	**96.81**	1.09	8.55	2.18
LONCHO	1.96	1.83	0.96	1.03	0.76	3.18	**97.24**	6.34	2.52
SENNOV	0.31	0.22	0.50	0.29	0.28	0.25	1.32	**98.53**	2.80
TRIGO	0.99	0.41	1.85	0.58	0.82	0.64	0.38	0.76	**98.61**
YENN	0.47	0.69	0.85	0.41	0.33	0.50	0.31	0.37	0.84

Note: species codes are corresponded to those in Fig. 2. The diagonal elements (highlighted) are mean TDR values of intraspecific queries. The upper triangular elements are mean TDR values of interspecific queries, the lower triangular elements the corresponding standard errors. Species encoding taken from Hebertetal2004 (see also Fig. 2).

Of the entities studied within the *Astraptes fulgerator* complex, the Bayesian method of Nielsen and Matz [27] could identify four of the nine with very high power (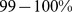) at the 0.05 significance level, while another three were identifiable with the power of 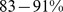. The power values for the two remaining groups INGCUP and HIHAMP were only 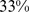 and 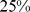. However, our TDR approach performed much better than did the Bayesian method. Averaging over the nine species tested, the mean power of the TDR approach was as high as 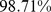, while the Bayesian approach obtained an average value of 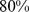 (Table 3). Significantly, in the cases for which the Bayesian method had most difficulty (the power was only 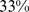 and 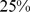 (INGCUP and HIHAMP)), our TDR approach achieved power values of 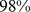 and 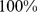. We also calculated the average probabilities of mis-assignment of each species to other species in the database, i.e. in pairwise comparisons. We found we had very slim chance (probability 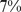, 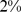 and 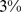) of mis-identifying any individual of species TRIGO, SENNOV, and YEN as other species. For those in species INGCUP, LOHAMP and LONCHO, the probabilities of mis-assignment increased to 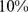, 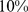, and 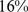. The worst cases occurred for HIHAMP, for which the probability of mis-assignment was as high as 30%. However, we note that in practice every mis-assignment of a HIHAMP sequence to another species would not lead to misclassification because it would also be assigned to HIHAMP with high probability (100%).

**Table 3:** Comparison of the performance of a Bayesian approach Nielsenetal2006 and the new TDR approach in species identification using Neotropical skipper butterfly species of the “*Astraptes fulgerator*” complex.

	Bayesian approach^a^		TDR approach	
	No. of sequences	Power^b^ in detecting conspecies	No. of sequences	Power in detecting conspecies ( 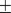 SE)
CELT	23	100	22	100.00 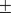 0.00
TRIGO	51	100	48	98.53 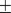 0.76
SENNOV	102	99	89	97.24 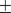 1.32
INGCUP	65	33	64	97.90 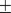 2.09
FABOV	31	91	29	99.33 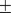 0.47
HIHAMP	16	25	14	100.00 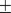 0.00
LOHAMP	47	83	42	100.00 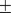 0.00
LONCHO	41	89	34	96.81 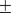 3.18
YENN	79	100	65	98.61 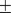 0.84
Average		**80**		**98.71**

a : the values were taken from Table 1 in Nielsenetal2006.

b : the average power is obtained by 10 replications (see also text).

#### Correlation analysis

To investigate the dependence of TDR values on molecular evolutionary models, we performed a correlation analysis for TDR values which are based on different evolutionary models (K2P versus HKY, both currently implemented in the software we are making available). First, we calculated TDR values based on both K2P and HKY genetic distances for coalescent simulated datasets (1 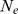, 5 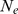, 10 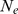 and 30 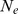) and the Neotropical skipper butterfly dataset. Second, correlation analysis of TDR values between K2P and HKY genetic distances were conducted. Weak or no statistical correlation between TDR values based on different evolutionary models would indicate a strong dependence of TDR values on the underlying evolutionary models, while a strong statistical correlation of TDR values obtained with different evolutionary models would suggest robustness of TDR values to the choice of evolutionary models, which would be an advantage of this newly defined measure of confidence. Strong and statistically significant correlations were observed between different evolutionary models for TDR values (R = 0.9607 0.9990 for 1 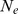, 5 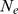, 10 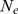 and 30 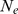, 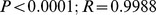 for the empirical data, 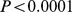;) (Figs. 3a–i). These results show that TDR values do not rely on the choice of evolutionary models. In the situation of shallow species trees (1 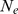 and 5 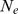), TDR values of interspecific query and intraspecific query overlapped due to incomplete lineage sorting (Figs. 3a,b,e,f). However, for deep species trees of 30 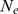, TDR values of interspecific queries and intraspecific queries were clearly separated as two clusters (Figs. 3d,h). This result demonstrates that species with a long divergence time relative to gene coalescence can be identified easily from each other through DNA barcoding. Two distinct clusters were also found for the empirical data set, with the exception of HIHAMP where a limited number of sequences (n = 14) was available. These data suggest that a small sample size of intraspecific reference sequences strongly increases the risk of false positives (see also Ross et al. [25]).

#### Estimating the rate of false positives with random sequences

Above we have reported the rates of false positives using the TDR approach under different coalescent simulation models (1 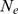, 5 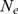, 10 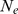 and 30 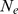, two effective population sizes 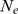
1 and 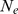
2) and with nine species of Neotropical skipper butterflies [33]. Here we investigate how the TDR procedure performs with randomly produced DNA query sequences. The reference dataset consisted of sequences from 32 simulated species (1 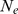, 5 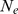, 10 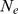 and 30 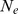) and nine real species, resulting in 41 datasets of reference sequences. Then, 820 randomly produced query DNA sequences of the same length as the reference sequences (648 bp) were generated. These 820 query DNA sequences were randomly divided into query groups corresponding to 41 datasets of reference sequences, each group containing twenty query sequences. TDR values were calculated according to formula (1) for each of 41 species, and mean TDR values over 20 queries were further summarized in Appendix S4. Both individual and average TDR values over 20 queries were very small (less than 0.05) for all 32 simulated species (Appendix S4), providing evidence for the absence of false positives when randomly generated sequences are considered. For the nine real species, six of them achieved extremely low mean TDR values (less than 1% against the maximum theoretical value of 100% for full confidence) with 20 random queries, whereas the remaining three resulted in moderately higher TDR values (27.30% for CELT, 12.05% for FABOV and 11.06% for HIHAMP; species code taken from Hebert et al. [33]) which are, nevertheless, far below the 95% confidence limit. This could be explained by their relatively small sample sizes (22, 28, 14, respectively) while the other six species have large sample sizes from 34 to 88 (Fig. 1, Appendix S4). With such small TDR values we would in practice always reject the conspecifity of randomly generated sequences with a given real species.

#### Sampling issue in DNA barcoding

**Figure 4:**
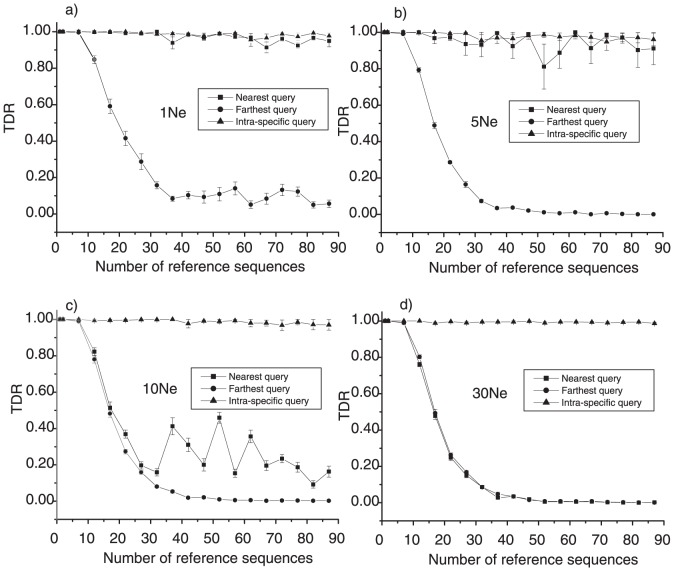
Correlation analysis of TDR values obtained with two different evolutionary models (HKY and K2P) for the coalescent simulated datasets (1 Ne, 5 Ne, 10 Ne, and 30 Ne) and examples of real species of Neotropical skipper butterflies. Solid squares in dased ovals show interspecific queries, and empty circles in vertical ovals with solid line indicate intraspecific queries. (a–d) correlation of TDR values between HKY and K2P evolutionary models in the situation of effective population size of 10,000 and with four tree depths (1 Ne, 5 Ne, 10 Ne, and 30 Ne). (e–h) correlation of TDR values between HKY and K2P evolutionary models in the situation of effective population size of 100,000 and with four tree depths (1 Ne, 5 Ne, 10 Ne, and 30 Ne). (i) Correlation of TDR values between HKY and K2P evolutionary models using empirical data of Neotropical skipper butterflies.

How many specimens per species should be sequenced in order to create a reliable reference database for species identification with DNA barcodes? The question has interested researchers from the beginning of the DNA barcoding initiative. All previous analyses found a curvilinear relationship of improvement in success with increasing number of reference sequences [25], but the threshold for reliable identification in practice has been controversial. Matz and Nielsen [26] proposed a number of 12 individuals per species while Ross et al. [25] advised that five or more reference sequences suffice to achieve good identification success. In an attempt to address this issue, we randomly chose one species as reference species from a species tree, and selected its nearest neighbor species (with the shortest average genetic distance) and the most distantly related species (with the largest average genetic distance) as query species on the same evolutionary tree. We called the former “Nearest query”, the later “Farthest query”. We simulated 800 sequences (100 for each of eight species) on the species tree. Eighteen different sizes of reference sequences from 2 to 87 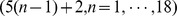 were used for all simulations with four tree depths (1 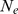, 5 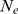, 10 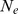 and 30 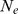). Ten query sequences from each of the reference species, the nearest query and the farthest query were randomly chosen. The average TDR values over ten queries with different sizes of reference sequences are presented in Fig. 4. In all simulated scenarios, the average intra-specific TDR values were almost close to 100%, even with a low number of reference sequences (Figs. 4a–d). This result demonstrates the low probability of false negatives when using TDR values as a measure of confidence of species membership in DNA barcoding, i. e. when a query sequence really does belong to a species, it is almost impossible for it to be assigned to the wrong species. With an increasing number of reference sequences, the farthest queries showed gradually decreasing average TDR values, which means that a large set of reference sequences will lower the risk of false positives. If we consider a TDR value of 95%. as a threshold of conspecifity, we found a sample size of 12 sufficient to avoid wrong assignments of the farthest species to the reference species in all evolutionary scenarios. In the cases of relatively deep species trees (10 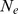, 30 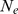; Appendix S1b:c,d), a set of 12 reference sequences also seems large enough to avoid wrong assignments (false positives) of the nearest query species to the reference species. However, with shallower tree depths (1 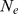, 5 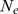, Figs. 4a,b), it was very hard to distinguish the nearest species from reference species even with large sets of reference sequences (87 individuals per species). We conclude that species identification in general suffers from a high risk of false positives with less than 12 reference sequences per species. However, when species paraphyly and incomplete lineage sorting are of concern, as envisioned in situations of shallow divergence combined with high effective population sizes, even with more than 12 reference sequence a reliable identification is hardly feasible. Seeking a universal size of reference sequence sets does not seem to be meaningful without taking the evolutionary history of individual species groups into account.

### Processing time

For each query, the run time largely depends on the length of sequences and the number of individuals of the potential species to which the query belongs. The data analyses in this study were performed on a 3.00 GHz desktop computer (Intel(R) Core (TM)2, DuoCPU, E8400 @ 3.00 GHz×2). The TDR approach spent average 32.38 seconds per query on the windows system, depending on the dataset size (for example, 288 reference sequences, each species containing 24 reference sequences in our simulated data), requiring 787 Mb RAM and 613 Mb virtual memory for a simulated data set.

## Materials and Methods

### Confidence Estimation Under DNA Barcoding—Null Hypothesis

Assume a population of a known species from which individuals were randomly sampled for a DNA barcoding project, with this set then defining species membership. Furthermore, let there be an unknown query sequence which may or may not belong to this particular species according to a minimum genetic distance or any other criterion. The null hypothesis used here is that the unknown query sequence is assumed to belong to the population of the known species when enough evidence isn't found to support that the query sequence is from a different species.

### Definition of confidence of species membership—TDR value

TDR is defined as the probability of a query sequence belonging to a group of reference sequences (predefined species),
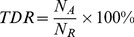
(1)where, 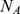 is the number of cases where the null hypothesis that a query sequence belongs to a group of reference sequences (see below) is accepted during vertically random resampling individuals from the pooled reference and query sequences, and 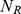 is the total number of replications of horizontal random resampling over sites along the sequences.

### Calculation of TDR with a 2D non-parametric resampling approach

**Figure 5:**
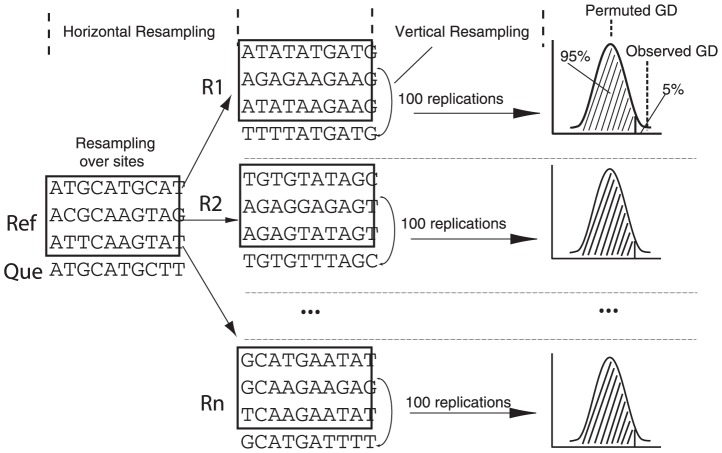
Mean TDR values based on different numbers of reference sequences. (a) TDR values computed on 1 depth species tree. (b) TDR values computed on 5 depth species tree. (c) TDR values computed on 10 depth species tree. (d) TDR values computed on 30 depth species tree. Triangles mean TDR values of intraspecific query. Squares indicate TDR values of nearest query and solid circles mean TDR values of farthest query. The horizontal bars above and below triangles, squares or solid circles indicate standard errors over 10 replications (queries).

TDR is estimated by a two-dimensional non-parameteric resampling approach Fig. 5. First, all reference sequences and the query are pooled into a sequence matrix 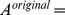


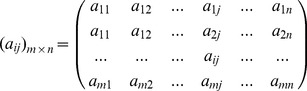
(2)

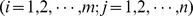
 where, 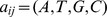, 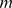 is the total number of reference and query sequences, 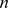 is the number of sites (characters) along the barcoding gene.

Second, the matrix 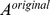
(2) is horizontally resampled over sites along the sequence by randomly selecting one character (column) from the original matrix to join a new matrix 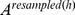 until the latter has as many characters as the original 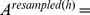


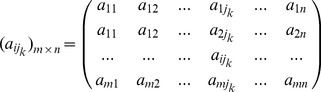
(3)where, 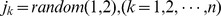, is a random integer in the range of 1 to 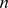. This step is identical to the initial step of a common bootstrapping analysis (CBA) in phylogenetic analyses, but next we conduct a vertical resampling over individuals instead of phylogenetic reconstruction.

Third, to take population sampling errors into account, a vertical (i.e., between individuals) random permutation over the matrix 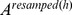
(3) is performed by randomly designating a sequence in 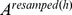 as the query sequence, and the remaining sequences as reference sequences. The randomly chosen query sequence is stored in a matrix
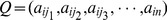
(4)where, 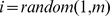 is a random integer in the range of 1 and 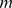, and 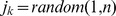 is a random integer between 1 and 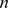. The remaining (reference) sequences are stored in a matrix 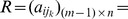


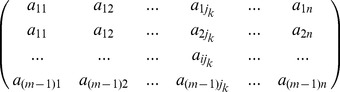
(5)


The genetic distance between the randomly-designated query sequence and the reference sequences is then determined as the mean of the distances between it and every reference sequence:
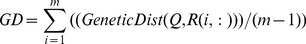
(6)where the function 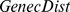 is currently implemented for an uncorrected genetic distance (absolute nucleotide differences - the p distance) and two corrected genetic distances - K2P distance [35] and HKY distance [30]. This process of randomly choosing a sequence from the set to be the designated query sequence is repeated a large number of times, say 100 but preferably over 1000 times, yielding a distribution of mean genetic distances under the hypothesis that the actual query sequence belongs to the test species. When the genetic distance between the actual query sequence and the actual reference sequences falls within the lower 95% of the simulated distribution, the null hypothesis is accepted, otherwise it is rejected (Fig. 5). 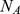 is defined as the number of times the null hypothesis is accepted in all 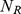 simulated datasets (produced by horizontal resampling).

We now examine the degree of bias and robustness of this simple measure in estimating confidence in specimen identification under DNA barcoding. Based on the definition in equation (1), we can easily derive the following useful expressions:
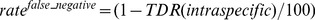
(7)where 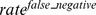 is the possibility when a query actually belongs to a specific species but rejected in error, and 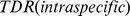 is a TDR value based on an intraspecific query.
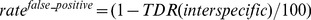
(8)where 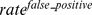 is the possibility when a query does not belong to a specific species but accepted in error, and 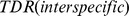 is a TDR value based on an intraspecific query.
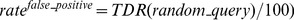
(9)where 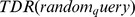 is a TDR value based on a specific interspecific query using a randomly generated sequence.

## Discussion

We have developed a method called Two Dimensional non-parametric Resampling (TDR) for measuring confidence of species membership using DNA barcoding. Pioneering attempts to introduce statistical approaches into DNA barcoding were made by Matz and Nielsen [26] and Nielsen and Matz Nielsenetal2006. However, as noted by these authors themselves [27], the proposed Bayesian method for DNA barcoding is difficult to apply. Besides the restrictive population genetic assumptions behind these methods, complexity in demanding computation has also prevented wide use of their approaches in the practice of DNA barcoding. Furthermore, these previous statistical studies on DNA barcoding did not give a direct measure of confidence of species membership, noted as highly desirable by Moritz and Cicero [17]. In this study, we developed such a direct measure of confidence of species membership - the TDR value. In contrast to the Bayesian and approaches, the TDR method stands out due to simplicity in both concepts and calculations, with little in the way of restrictive population genetic assumptions. We have demonstrated its power in measuring confidence with coalescent simulated datasets in different evolutionary scenarios (four tree depths, combined with two different effective population sizes) and an empirical dataset. We found our theoretical expectations widely confirmed. Regardless of the underlying species tree, almost all intraspecific queries 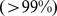 fell within the 95% confidence limit 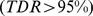, while neither randomly simulated sequences nor interspecific sequences on deep and moderately deep species trees (5 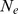, 10 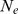, 30 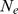) reached statistical significance. Only in the case of very shallow divergence (1 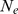) is the outcome more ambiguous, with a slightly higher probability 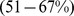 of false positives. Similar conclusions can be drawn from the empirical example analyzed of Neotropical skipper butterflies. Therefore we suggest that TDR values could yield an effective and robust measure of confidence in DNA barcoding projects.

A comparison of detecting power in species identification was performed between TDR approach and the Bayesian method through the empirical example of the Neotropical skipper butterfly “*Astraptes fulgerator*” species complex. The TDR approach performed markedly better than did the Bayesian method, especially in the extremely difficult cases where the latter found out the correct species with only 33% and 25% power respectively (INGCUP and HIHAMP) while the TDR approach performed correct species identification with the power of 98% and 100% correspondingly. More comparisons with empirical examples are desirable to further test our conclusion since we only made a comparison through a Neotropical skipper butterfly example mainly due to the lack of easy-to-use publicly available computer program of the Bayesian method [10]. A simple BLAST approach [36], [37] has been criticized for potential false assignment). A tree-based genetic distance approach (such as using the Neighbor Joining method, [1], [2]) will require correct taxa represented in the users' reference data base otherwise wrong assignment will happen. These approaches generally use a raw similarity scores to produce a nearest neighbor that is not necessarily the closest relative [38]. For a new DNA barcoding sequencing project, researchers will not know whether the species they are sequencing are already in the database or not. The TDR approach proposed here will not necessarily require query sequences to have representative sequences in the database; TDR values mainly serves as a measure of statistical confidence in species assignment similar to bootstrap values in phylogenetic analyses. Incomplete lineage sorting and hybridization are two very important evolutionary processes that have been well-documented [39]–[44]. Especially, some statistical approaches for distinguishing between them have been proposed [40], [41]. However, the power of the test was effectively best only when sequences had a length of 5,000 bp which is almost impossible for the current worldwide DNA barcoding campaign [41], indicating the difficulty of detecting hybridization from incomplete lineage. Furthermore, the currently used short segment of COI gene (648 bp or so) for animals will suffer from maternal hereditary of mtDNA, where correct assignments via COI DNA barcoding are largely based on congruence between gene tree and species tree. The current version of TDR approach is unable to distinguish incomplete lineage sorting from hybridization. However, it's expected that applying TDR approach to multiple barcodes will shed some lights on this issue.

Another limitation of the method is the restriction to DNA sequence data, but other characteristic systems, such as morphological and behavior characteristics could be incorporated in the future [12], [45]. In DNA barcoding practice, the application of the TDR measure may be also limited by insufficient and non-random intraspecific sampling, which is a common problem for all statistical inferences in DNA barcoding. At the current stage of development of DNA barcoding databases, the depth of individual species sampling is usually sacrificed in favor of greater taxonomic coverage [26]. For example, the database of DNA barcoding (http://www.barcodinglife.org/views/login.php) includes typically just five to ten sequences for the majority of species ([46], see also http://www.barcodinglife.org/views/login.php) with a high proportion of singletons. To reduce the risk of false positive inference, we suggest a minimum sample size of 12 individuals per species, as proposed by Matz and Nielsen [26], although looking for a universal value of reference sampling size seems problematic without taking evolutionary histories into account. We agree with Matz and Nielsen [26] on calling for a balance between intraspecific and interspecific sampling.

Besides the advantages mentioned above, the TDR measure has additional favorable characteristics, such as taking both gene and population sampling errors into account, relative independence from evolutionary models, and providing an independent line of evidence in addition to other methods, such as MP, Bayesian, or BP-based species identification in DNA barcoding [12]. We postulate that the TDR measure also has potential for species discovery in DNA barcoding projects when applied in combination with artificial-intelligence based methods of species identification.

Finally, for on-going DNA barcoding practice, we strongly propose (1) to distinguish between the specimens which are identified through morphology (predefined) and those identified via DNA barcoding; (2) to add more collateral information for the DNA barcoded specimens, such as genes applied (e.g. cox1 currently used), methods used for identification (NJ, MP, BP-based, and others), a measure of confidence for that assignment (e.g. TDR measure proposed in this study). All this information could be incorporated into the current database (http://www.barcodinglife.org/views/login.php). For example, a query sequence identified to a predefined species A, could be recorded as 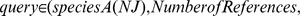 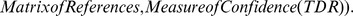


## Conclusion

TDR measure provides biologists a simple and direct method to assess confidence of species membership in DNA barcoding without introducing restrictive population genetive assumptions, and demanding computation. The corresponding computer program (TDR1.0beta) has been developted, and will be very helpful to biologists in the field of DNA barcoding.

## Supporting Information

Appendix S1 **Species tree topologies and gene trees.** (a) Different species tree topologies used in this study and (b) Examples of gene trees (white, inside) simulated by neutral coalescence within simulated species trees (black, outside) in the coalescent simulation scenario.
(TIF)

Appendix S2 **TDR values simulated on 10 different species trees with different settings of parameters.**
(TXT)

Appendix S3 **Interspecific and intraspecific TDR values simulated with additional settings of parameters,including two more values of transition/transversion ratio (0.5 and 13), gamma parameter (0.2 and 20).**
(JPG)

Appendix S4 **TDR values of random queries over simulated and empirical species.**
(JPG)
