# Supplementary figures and images for: A Simple 2D Non-Parametric Resampling Statistical Approach to Assess Confidence in Species Identification in DNA Barcoding—An Alternative to Likelihood and Bayesian Approaches
Source: PLoS One. 2012 Dec 11;7(12):e50831. doi: 10.1371/journal.pone.0050831 (PMC3519818; doi:10.1371/journal.pone.0050831)

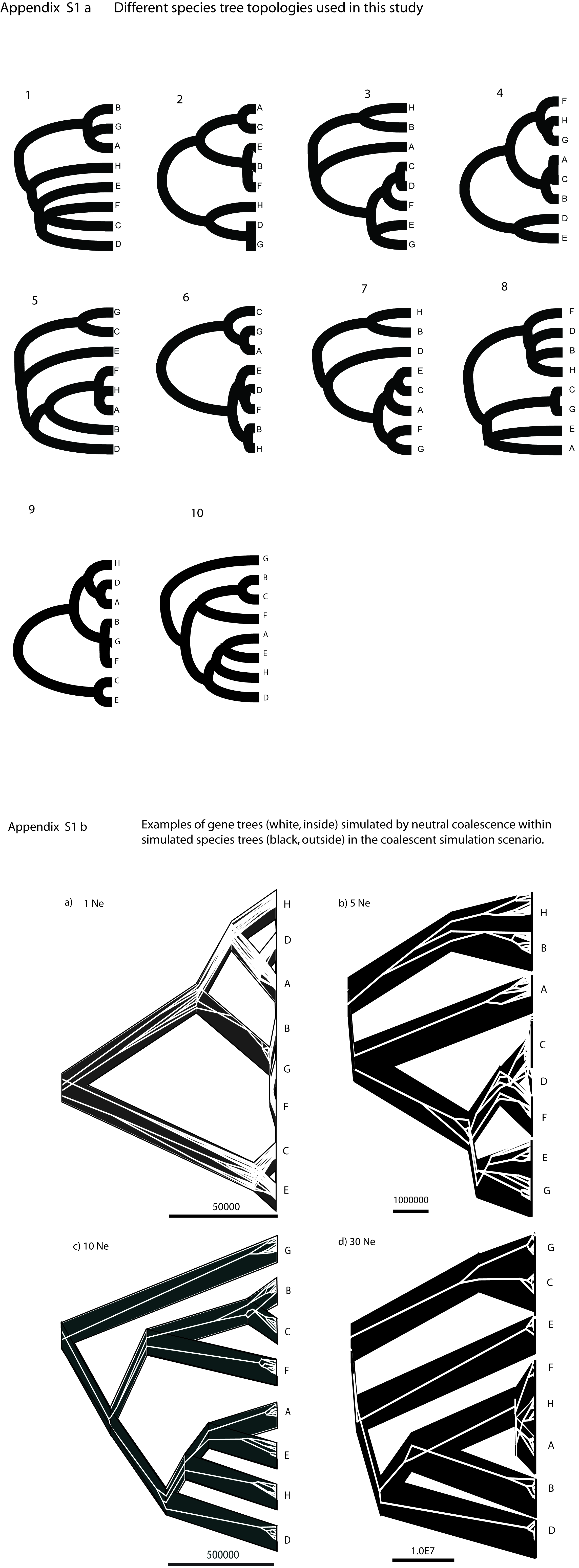

Supplement: Appendix S1 — Species tree topologies and gene trees. (a) Different species tree topologies used in this study and (b) Examples of gene trees (white, inside) simulated by neutral coalescence within simulated species trees (black, outside) in the coalescent simulation scenario. (TIF) [file pone.0050831.s001.tif]

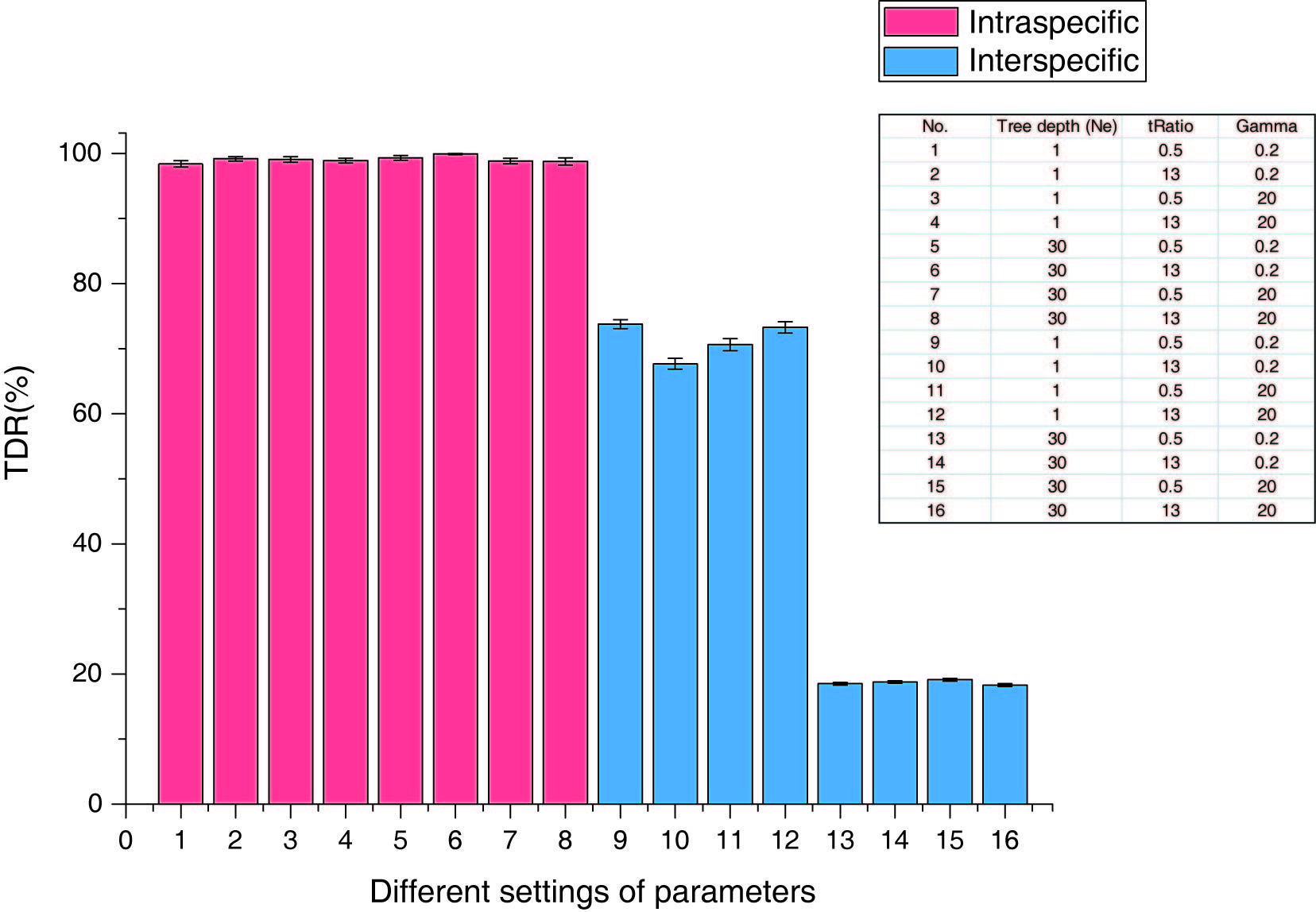

Supplement: Appendix S3 — Interspecific and intraspecific TDR values simulated with additional settings of parameters,including two more values of transition/transversion ratio (0.5 and 13), gamma parameter (0.2 and 20). (JPG) [file pone.0050831.s003.jpg]

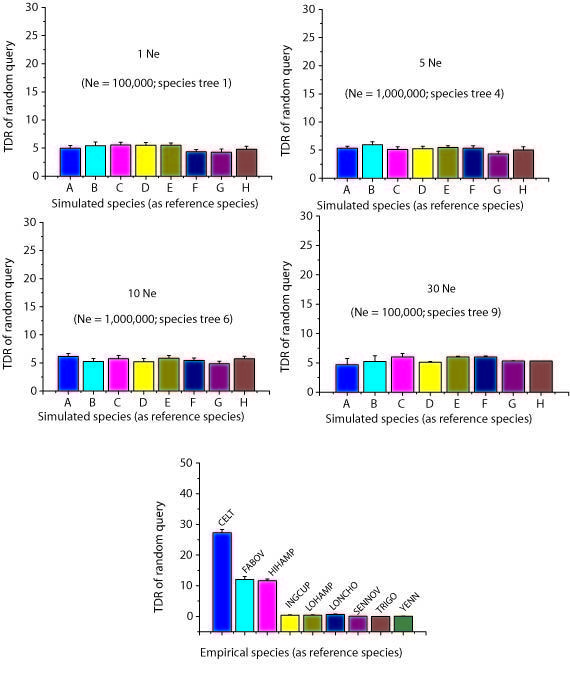

Supplement: Appendix S4 — TDR values of random queries over simulated and empirical species. (JPG) [file pone.0050831.s004.jpg]
